# New special approach for shoulder stability after Malawer type IVB shoulder girdle resection: A case report

**DOI:** 10.1016/j.ijscr.2022.107189

**Published:** 2022-05-11

**Authors:** T. Furuta, T. Sakuda, K. Oae, Y. Harada, K. Arihiro, N. Adachi

**Affiliations:** a Department of Orthopaedic Surgery, Hiroshima University, Graduate School of Biomedical and Health Sciences, Hiroshima, Japan; b Department of Musculoskeletal Traumatology and Reconstructive Surgery, Graduate School of Biomedical and Sciences, Hiroshima, Japan; c Department of Pathology, Hiroshima University Hospital, Hiroshima, Japan

**Keywords:** DASH, Disabilities of Arm, Shoulder, and Hand, MRI, magnetic resonance imaging, MSTS, Musculoskeletal Tumor Society Score, Malignant bone tumor, Scapulectomy, Shoulder girdle resection, Devices for shoulder stability, Malawer technique type IVB

## Abstract

**Introduction and importance:**

Scapular prostheses are useful in shoulder stability after shoulder girdle resection for malignant bone tumors; however, they are difficult to obtain in Japan. Therefore, other methods must be considered, depending on the extent of resection. We report a case in which a clavicle-locking plate, Nesplon tape, and a proximal humeral prosthesis were used to ensure shoulder stability and preserve stable upper limb function.

**Case presentation:**

A 56-year-old man presented with a large mass and edema over the entire right scapula, which caused severe pain, limited the shoulder's range of motion, and impaired function of the entire upper extremity. Clinical imaging and pathological findings indicated a diagnosis of conventional chondrosarcoma. Using the Malawer technique type IVB, we resected the shoulder girdle and secured shoulder stability with a clavicle-locking plate, Nesplon tape, and a proximal humeral prosthesis. To evaluate the patient, we obtained his Musculoskeletal Tumor Society (MSTS) and Disabilities of Arm, Shoulder, and Hand (DASH) scores 3 months postoperatively.

**Clinical discussion:**

To preserve the function of the patient's elbow and hand, the stability of his shoulder was important. We could achieve this stability by using a prosthesis available in Japan. The patient's MSTT and DASH scores improved remarkably.

**Conclusion:**

A clavicle-locking plate, Nesplon tape, and a proximal humeral prosthesis can be used to ensure shoulder stability after scapular girdle resection and can preserve or improve upper limb function.

## 1 Introduction

Although malignant bone and soft-tissue tumors of the upper extremities are less common than those of the lower extremities, the scapula and proximal humerus are typical sites of primary sarcomas (osteosarcoma, Ewing's sarcoma, and chondrosarcoma) [1], [2]. Osteosarcoma and Ewing's sarcoma are commonly managed with adjuvant treatment, such as chemotherapy and radiation therapy, in combination with surgical treatment, whereas for chondrosarcoma, surgery is the first choice of treatment.

Until the late 20th century, forequarter amputation was the standard treatment for malignant tumors of the shoulder girdle; since then, most malignant tumors of the shoulder girdle have been treated with limb-sparing resection. However, this procedure causes large bone and soft-tissue defects in the shoulder girdle. For soft-tissue defects, a latissimus dorsi skin flap is often used for soft tissue defect because of its anatomical advantages [3], [4], [5]. Various reconstruction methods have been reported for bone defects, depending on the extent of the resection: results have been good when the glenohumeral joint is spared but only moderate when the scapula and proximal humerus cannot be spared [6].

Our patient had a conventional chondrosarcoma of the scapula that extended to the articular surface; therefore, we used the Malawer technique type IVB (removal of the entire scapula, proximal humerus, and part of the abductor muscles) for resection. Reconstruction of the bone defect was challenging; we tried to obtain a scapular prosthesis, as advocated in some reports, but it was impossible to order one in Japan [7], [8], [9]. Nonetheless, after shoulder girdle resection, it was necessary to devise a way to achieve shoulder stability and to prevent significant functional impairment of the upper limbs.

In previous case report, the clavicle and humerus were cemented with a pedicle screw system to ensure shoulder stability after shoulder girdle resection; however, the strength of the screw was questionable, and we judged that it would soon fracture if it could not withstand rotational movements [10]. Instead, we used a clavicle-locking plate (VariAx 2 Clavicle Plating System; Japan Striker Corp., Tokyo, Japan), Nesplon tape made of polyethylene (Alfresa Pharma Corp., Osaka, Japan), and a proximal humeral prosthesis available in Japan (Aequalis Fracture; Japan Striker Corp., Tokyo, Japan). Preoperative and postoperative radiographic and functional evaluations were performed, and the Musculoskeletal Tumor Society (MSTS) score and the Disabilities of Arm, Shoulder, and Hand (DASH) score were obtained to verify whether this method ensured shoulder stability after shoulder girdle resection [11], [12]. Treatment interventions were performed in compliance with SCARE 2020 [13].

## 2 Presentation of case

**Fig. 1:**
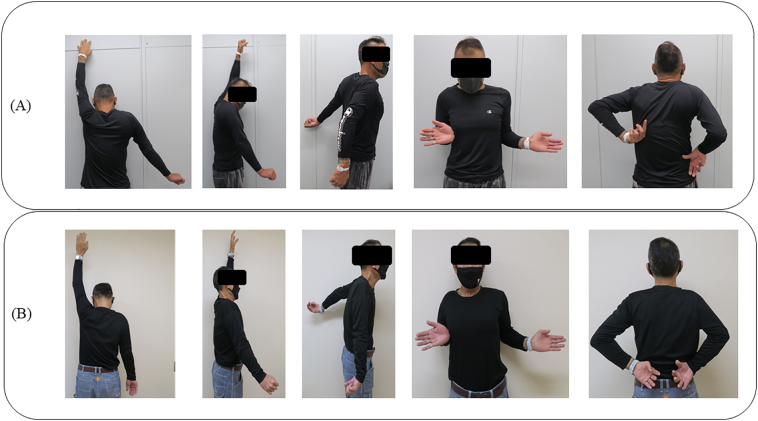
Range of motion of the repaired shoulder joint. (A) Preoperative range of motion. Right to left: abduction, flexion, extension, external rotation, and internal rotation. (B) Range of motion 3 months after surgery. Right to left: abduction, flexion, extension, external rotation, and internal rotation.

**Fig. 2:**
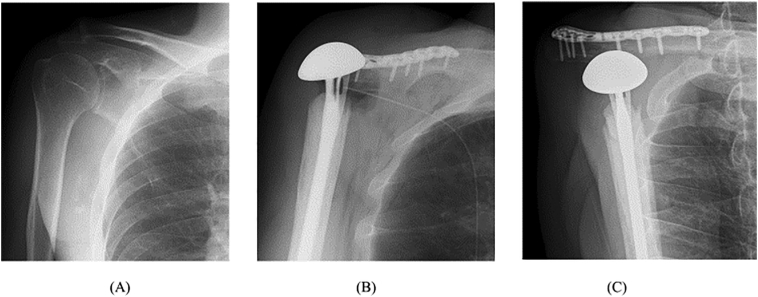
Preoperative and postoperative radiographs of a 56-year-old man's right shoulder. (A) Preoperative frontal view with suspected osteolysis of the scapula. (B) Frontal view after shoulder girdle resection, with shoulder stability provided by the clavicle-locking plate, Nesplon tape, and a proximal humeral prosthesis. (C) Postoperative frontal view implant head slightly more drooped than immediately after surgery, but stable.

**Fig. 3:**
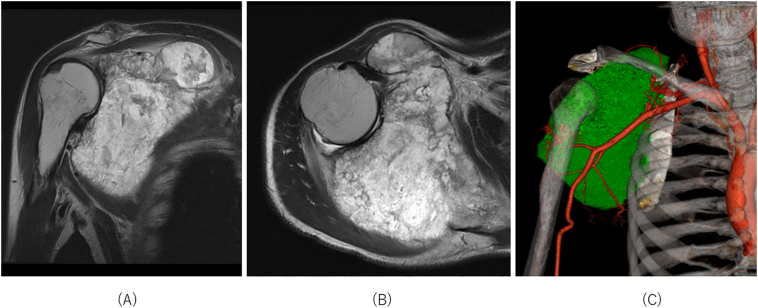
Preoperative images of the malignant scapular tumor in the right shoulder. On magnetic resonance imaging, the frontal view of the right shoulder (A) showed malignant tumor invasion of the entire scapula and glenoid fossa but not the acromioclavicular joint. The axial view of the right shoulder (B) showed malignant tumor invasion of the deltoid muscle (abductor muscle). Three-dimensional computed tomographic angiography (C) showed several feeding vessels extending from the axillary artery to the malignant tumor.

A 56-year-old man, a manual laborer, developed a mass in the right shoulder girdle 2 months ago, accompanied by marked pain, swelling of the right upper extremity, and dysfunction of the right elbow and hand. He was diagnosed with scapular malignancy by Magnetic resonance imaging (MRI) by his previous physician and referred to the Department of Bone and Soft Tissue Oncology at our hospital. A physical examination revealed a huge, elastic, hard mass at the shoulder girdle. The entire right upper limb was edematous, and the range of motion of the right shoulder joint was markedly limited. Abduction was 45° right and 170° left, flexion was 20° right and 170° left, extension was 10° right and 30° left, external rotation was −5° right and 50° left, and internal rotation was Th11 level on the right and Th6 level on the left (Fig. 1A). Marked edema and pain restricted the use of his right elbow and hand in daily life. The patient's MSTS score was 3 (10.0%) and the DASH score was 87.06. Radiographs depicted osteolysis of the right scapula and mass shadow (Fig. 2A). MRI showed a large tumor with heterogeneous T1 isointensity and high T2 intensity. The tumor had not invaded the acromioclavicular joint, but it had invaded the supraspinatus, infraspinatus, and subscapularis muscles, and parts of the deltoid muscles (Fig. 3A, B). Three-dimensional computed tomographic angiography showed that the tumor had a feeding artery and was adjacent to but had not invaded the subclavian and axillary arteries (Fig. 3C).

**Fig. 4:**
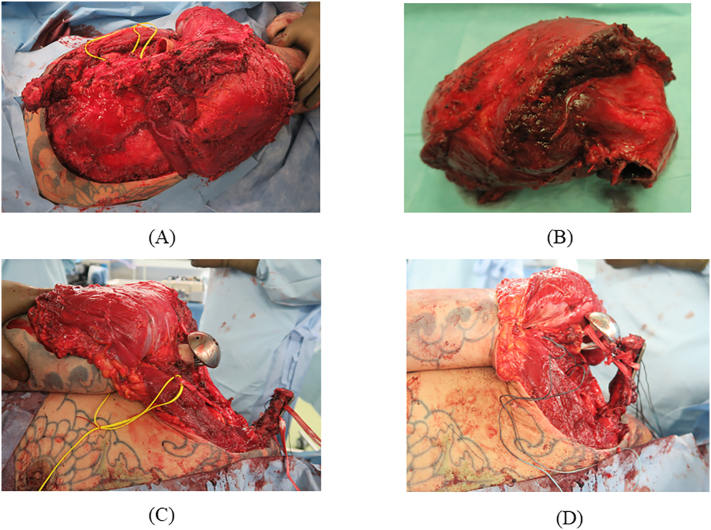
Intraoperative photographs. (A) The Malawer technique type IVB was used to remove the entire scapula, the proximal humerus, and part of the abductor muscles, but the clavicle was preserved. (B) The tumor was widely resected. (C) The head of the proximal humeral prosthesis was placed at an inverted 180° angle to avoid pressure on the axillary artery and brachial plexus, in contrast to the instructions in the product manual. Nesplon tape was fixed tightly to the clavicle and clavicle-locking plate so that the tape was sandwiched between them. (D) A clavicle-locking plate, Nesplon tape, and a proximal humeral prosthesis were used to achieve shoulder stability.

Needle biopsy was performed, and chondrosarcoma was diagnosed. Surgery was the first choice of treatment. We planned to use the Malawer technique type IVB to resect the scapula, proximal humerus, rotator cuff, and parts of the deltoid muscles en bloc. All surgeries were performed by T. Furuta, Bone and Soft Tissue Oncology Surgery, and K. Oae, Trauma and Bone Reconstruction Specialist. First, the entire scapula was exposed, and the tumor invasion area of the deltoid muscle was attached to the tumor side. The axillary artery, axillary vein, and brachial plexus were identified and preserved. The myocutaneous nerve was identified and preserved, the proximal humerus was osteotomized, the acromioclavicular ligament was separated, and the scapular girdle was resected along with the malignant tumor (Fig. 4A, B).

Next, Nesplon tape was fixed tightly to the clavicle and clavicle-locking plate so that the tape was sandwiched between them. When the proximal humeral prosthesis was placed as described in the product instruction manual and connected with Nesplon tape, the head of the prosthesis compressed the axillary artery and axillary vein and brachial plexus. We rotated the head of the prosthesis 180° and re-placed it; the prosthesis was thereby stabilized without compressing the neurovascular bundle. The prosthesis was cemented in place, and it and the clavicle were connected with Nesplon tape (Fig. 4C, D). Radiographs confirmed the stability of the shoulder (Fig. 2B).

In addition, the biceps, triceps, and coracobrachialis muscles were attached to the prosthesis. The portion of the deltoid muscle that could be preserved was sutured to the trapezius muscle.

A pathological evaluation of the resected tumor revealed conventional chondrosarcoma, and the resection margins were negative. Marked improvement in right shoulder joint internal rotation movement was observed: Abduction was 20° right and 170° left, flexion was 25° right and 170° left, extension was 5° right and 30° left, external rotation was 0° right and 50° left, and internal rotation was Th7 level on the right and Th6 level on the left (Fig. 1B).

**Fig. 5:**
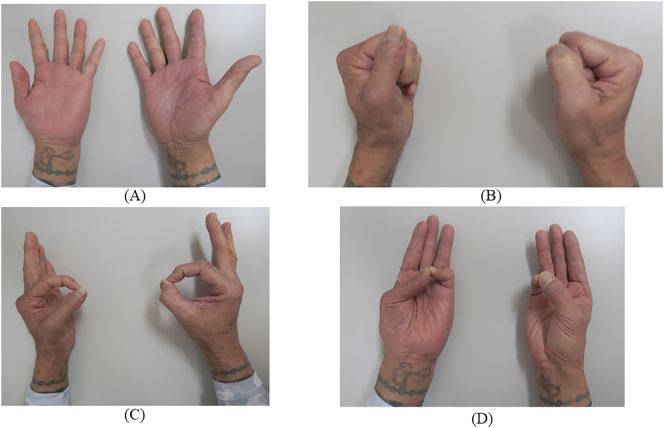
Hand movement evaluation 3 months after surgery. The left side is the uninjured hand, and the right side is the repaired hand. (A) Extension of the hand. (B) Flexion of the hand. (C) Pinching motion of the thumbs and index fingers. (D) Opposing motion of the thumbs and little fingers.

Three months after surgery, radiographs show that the bone head of the implant has been slightly lowered and stabilized (Fig. 2C), and the patient's MSTT score was 22 (73.3%) and the DASH score was 60.03, indicating marked improvement in right upper limb function (Fig. 5). Patient has returned to work with some exercise restrictions and been very satisfied. The patient had a good understanding of the therapeutic intervention, was able to comply with our instructions, and had no problems after the intervention.

## 3 Discussion

Surgery for malignant bone and soft-tissue tumors of the shoulder girdle involves three stages: (1) surgical resection, based on oncological concepts; (2) reconstruction for bone defects, and (3) reconstruction for defects of soft tissue, such as skin and muscle. Our patient's conventional chondrosarcoma had invaded the entire scapula, the glenoid fossa, and part of the abductor muscle group but not the acromioclavicular joint; therefore, the clavicle could be preserved, and we used the Malawer technique type IVB for the surgery [10], [14]. The tumor did not invade the skin, and the muscle involvement allowed preservation of part of the deltoid and trapezius muscles, so soft tissue reconstruction was not necessary. The major problem in this case was the reconstruction of bone defects. Endprosthetic replacement to stabilize the glenohumeral joint is common [7], [8], [9], [14], but we could not obtain such a prosthesis in our country. To preserve the function of the elbow and hand [15], we considered using a free vascularized fibula graft to connect the humerus to the clavicle, but damage to the patient's leg would have hampered his work as a manual laborer. The patient's clavicle had not been invaded by the tumor and could be preserved, and thus, we planned to replace the proximal humerus with a prosthesis, connect the clavicle to the prosthesis with Nesplon tape, and fix both the clavicle and prosthesis with a clavicle-locking plate to keep the tape in place. The advantage of this method is that it is more tolerant of rotational movement than the pedicle screw system [10] because the two structures are connected with tape. In addition, the biceps, triceps, and coracobrachialis muscles were attached to the prosthesis, thereby preserving right upper limb function.

If fixation is not part of reconstruction, appropriate decisions must be made intraoperatively. Reconstruction after shoulder girdle resection should be flexible, depending on the extent of resection, implants, and the patient's condition and wishes. To avoid damage to the patient's lower extremities and improve the function of his elbow and hand, we devised a new reconstruction method.

Techniques of reconstruction after shoulder girdle resection vary, but good results have been reported mainly with prostheses or allograft reconstruction [2], [7], [14], [15], [16], [17], [18], [19]. A free vascularized fibular graft after shoulder girdle resection has also been reported to yield good results as a functional spacer and sling procedure [20], but at the expense of the fibula.

In glenohumeral joint defects in which clavicle preservation is possible, as in this case, a clavicle-locking plate, Nesplon tape, and a proximal humeral prosthesis can be used to achieve shoulder joint stability. In fact, the patient was extremely satisfied with the improvement in shoulder extension and internal rotation, as well as with the improvement in elbow and hand function (Fig. 5). One possible limitation of this strategy concerns the durability should be noted due to the short follow-up period.

## 4 Conclusion

After scapular girdle resection that affects the glenohumeral joint, a clavicle-locking plate, Nesplon tape, and a proximal humeral prosthesis can be used to ensure shoulder stability and to preserve or improve upper limb function.

## Provenance and peer review

Not commissioned, externally peer-reviewed.

## Sources of funding

No external funding was received for the publication of this paper.

## Ethical approval

No ethical approval was necessary for the treatment or investigation of this patient.

## Consent

Written informed consent was obtained from the patient for publication of this case report and accompanying images. A copy of the written consent is available for review by the Editor-in-Chief of this journal on request.

## Author contribution

Taisuke Furuta will contribute to the research concept and design, data collection, analysis and interpretation, oversight and leadership responsibility for planning and execution of research activities, and external mentoring of the core team. We would like to thank the following for their contributions to this work: Enago (www.enago.jp), for English language review. Tomohiko Sakuda contributed to the data collection of the study. Yohei Harada contributed to the data collection, research concept, and treatment design of the study.

Kazunori Oae contributed to the data collection, research concept, and treatment planning of the study. Koji Arihiro contributed to pathological diagnosis and data collection.

Nobuo Adachi contributed to the concept, supervision, and guidance of the study.

## Research registration

Research Registry unique identifying number (UIN):7823.

## Guarantor

Tomohiko Sakuda.

Yohei Harada.

Taisuke Furuta.

## Declaration of competing interest

The authors declare no conflicts of interest.

## References

[1] Kiss J., Sztrinkai G., Antal I., Kiss J., Szendrol M. (2007). Functional results and quality of life after shoulder girdle resections in musculoskeletal tumors. J. Shoulder Elb. Surg..

[2] Biazzo A., De Paolis M., Donati D.M. (2018). Scapular reconstructions after resection for bone tumors: a single-institution experience and review of the literature. Acta Biomed.

[3] Kim J.S., Lee J.S., Yoon J.O., Park J.B. (2009). Reconstruction of the shoulder region using a pedicled latissimus dorsi flap after resection of soft tissue due to sarcoma. J. Plast. Reconstr. Aesthet. Surg..

[4] Behnam A.B., Chen C.M., Pusic A.L., Mehara B.J., Disa J.J., Athanasian E.A. (2007). The pedicled latissimus dorsi flap for shoulder reconstruction after sarcoma resection. Ann. Surg. Oncol..

[5] Capanna R., Manfrini M., Briccoli A., Gherlinzoni F., Lauri G., Caldora P. (1995). Latissimus dorsi pedicled flap applications in shoulder and chest wall reconstructions after extracompartimental sarcoma resections. Tumori.

[6] Ozturk R., Arikan S.M., Togral G., Gungor B.S. (2019). Malignant tumors of the shoulder girdle: surgical and functional outcomes. J. Orthop. Surg..

[7] Teunis T., Nota S.P.F.T., Hornicek F.J., Schwab J.H., Lozano-Calderon S.A. (2014). Outcome after reconstruction of the proximal humerus for tumor resection: a systematic review. Clin. Orthop. Relat. Res..

[8] Manili M., Fredella N., Santori F.S. (2002). Shoulder prosthesis in reconstruction of the scapulohumeral girdle after wide resection to treat malignant neoformation of the proximal humerus. Chir. Organi Mov..

[9] Streitbuerger A., Henrichs M., Gosheger G., Ahrens H., Nottrott M., Wiebke G. (2015). Improvement of the shoulder function after large segment resection of the proximal humerus with the use of an inverse tumour prosthesis. Int. Orthop..

[10] Prabowo Y., Saleh R.F. (2021). Pedicle screw system reconstruction in shoulder resection type IV-total scapulectomy: a case report and short term follow up. Int. J. Surg. Case Rep..

[11] Enneking W.F., Dunham W., Gebhardt M.C., Malawar M., Pritchard D.J. (1993). A system for the functional evaluation of reconstructive procedures after surgical treatment of tumors of the musculoskeletal system. Clin. Orthop. Relat. Res..

[12] Harrington S., Michener L.A., Kendig T., Miale S., George S.Z. (2014). Patient-reported shoulder outcome measures utilized in breast cancer survivors: a systemic review. Arch. Phys. Med. Rehabil..

[13] Agha R.A., Franchi T., Sohrabi C., Mathew G., for the SCARE Group (2020). The SCARE 2020 guideline: updating consensus Surgical CAse REport (SCARE) guidelines. International Journal of Surgery.

[14] Malawer M., Shugarbaker P.H. (2001).

[15] Mayil Vahanan N., Mohanlal P., Bose J.C., Gangadharan R., Karthisundar V. (2007). The functional and oncological results after scapulectomy for scapular tumours: 2–16-year results. Int. Orthop..

[16] Q Yang J Li Z Yang X Li Z. Lin. Limb sparing surgery for bone tumours of the shoulder girdle: the oncological and functional results. Int. Orthop. 34: 869–75. 10.1007/s00264-009-0857-3 PMC2989017 19701633

[17] Pritsch T., Bickls J., Wu C.C., Squires M.H., Malawer M.M. (2007). Is scapular endoprosthesis functionally superior to humeral suspension?. Clin. Orthop. Relat. Res..

[18] Getty P.J., Peabody T.D. (1999). Complication and functional outcomes of reconstruction with an osteoarticular allograft after intra-articular resection of the proximal aspect of the humerus. J. Bone Joint Surg. Am..

[19] Rodi R.W., Ozaki T., Hoffmann C., Böttner F., Lindner N., Winkelmann W. (2000). Osteoarticular allograft in surgery for high-grade malignant tumours of bone. J. Bone Joint Surg. Br.

[20] Wada T., Usui M., Isu K., Yamawakii S., Ishii S. (1999). Reconstruction and limb salvage after resection for malignant bone tumour of the proximal humerus. A sling procedure using a free vascularized fibular graft. J. Bone Joint Surg. Br..

